# Navigating Uncertainty: Health Professionals' Knowledge, Skill, and Confidence in Assessing and Managing Pain in Children with Profound Cognitive Impairment

**DOI:** 10.1155/2016/8617182

**Published:** 2016-12-21

**Authors:** Bernie Carter, Joan Simons, Lucy Bray, Janine Arnott

**Affiliations:** ^1^Faculty of Health and Social Care, Edge Hill University, Ormskirk, UK; ^2^Faculty of Health and Social Care, The Open University, Milton Keynes, UK; ^3^College of Health and Wellbeing, University of Central Lancashire, Preston, UK

## Abstract

There is limited evidence to underpin the assessment and management of pain in children with profound cognitive impairment and these children are vulnerable to poor pain assessment and management. Health professionals working with children with profound cognitive impairment from a single paediatric tertiary referral centre in England were interviewed to explore how they develop and acquire knowledge and skills to assess and manage pain in children with cognitive impairment. The interviews were transcribed and subjected to thematic analysis. Nineteen health professionals representing different professional groups and different levels of experience participated in the study. A metatheme “navigating uncertainty; deficits in knowledge and skills” and two core themes “framing as different and teasing things out” and “the settling and unsettling presence of parents” were identified. Uncertainty about aspects of assessing and managing the pain of children with cognitive impairment tended to erode professional confidence and many discussed deficits in their skill and knowledge set. Uncertainty was managed through engaging with other health professionals and the child's parents. Most health professionals stated they would welcome more education and training although many felt that this input should be clinical and not classroom oriented.

## 1. Introduction

There are varied and interchangeable terms used within the literature to describe the diverse group of children who are so severely cognitively impaired that they are unable to self-report their pain as they lack the capacity to either verbally communicate or purposefully communicate their pain through other systems. These children are described as having special needs, intellectual disability, neurological disability, developmental disability, and cognitive impairment; the descriptors are often qualified by terms such as severe and profound to reflect the depth of disability or impairment [1]. The term profound cognitive impairment was used in this study as it reflected the depth of the cognitive impairment of the children in our study and it was the descriptor that our parent advisors preferred.

Children with profound cognitive impairment are a heterogenous group in terms of their underlying condition (e.g., birth asphyxia, cerebral palsy, and neurodegenerative and metabolic disorders) and the range of comorbidities they experience (e.g., seizure disorders, perceptual disorders, physical impairments, and respiratory and feeding problems) [2]. The interplay of each of these elements across diagnostic groups as well as within each child adds to the heterogeneity of this “group” and creates a particular challenge in terms of developing a robust evidence base about their pain. The current evidence base is not robust and it typically treats this heterogeneous group of children as homogenous and prioritises the profundity of impairment rather than taking full account of the interplay of diagnoses and comorbidities. Studies are often small scale, underpowered, and not adequately representative of the children's diverse diagnoses, comorbidities, capacities, and treatments. These limitations within the evidence base need to be considered in relation to the findings of the small number of relevant studies that are now reported.

Children with profound cognitive impairment have a higher number of nociceptive and neuropathic pain episodes compared to their healthy peers and these children can experience frequent and significant pain, sometimes on a daily basis [3–5]. Different approaches have been used to categorise the causes of pain [3, 4, 6] with agreement that the main sources of pain are musculoskeletal, gastrointestinal, infection, and iatrogenic. Evidence shows that the number of comorbidities increases with age [5]. Incidence studies are rare and Breau et al.'s study of the caregivers of 94 children with severe cognitive impairment, aged 3–18 years, is a milestone in identifying the range and extent of pain children with cognitive impairment experience [4]. This study found a high incidence of pain with 35%–52% (*n* = 33–49) of children in the sample experiencing pain for an average of 9-10 hours per week with a mean intensity of 6.1 (0–10 rating scale). However, despite similar findings from other studies showing that this diverse group of children experience regular and persistent pain [7, 8] it is clear that further research is needed to generate a clear understanding of the epidemiology of pain as the heterogeneity of children with profound cognitive impairment means that findings cannot easily or reliably be transferred.

There is limited evidence to underpin the assessment and management of pain in children with cognitive impairment [9] and less for children with profound cognitive impairment; this leaves these children particularly vulnerable to poor pain assessment and management. Studies have demonstrated that the parents of children with profound cognitive impairment often develop knowledge and skills experientially to determine whether their child is in pain [3, 6, 10–12] and are sensitive pain detectors [13], although they may also underestimate their child's pain [14]. Studies have shown that where parents receive information about and have access to a structured observation tool, their skills and confidence in assessing their child's pain increase [12] and others emphasise the importance of health professionals working in partnership with parents to improve the quality of pain assessment [15].

Health care professionals report a lack of confidence in undertaking pain assessment in children with cognitive impairment [6]. This is despite the fact that robust tools have been validated for use in children who lack the ability to verbally report pain due to profound cognitive impairment [16]. Appropriate tools include the Paediatric Pain Profile [5], the revised-Face, Legs, Activity, Cry, Consolability (r-FLACC) tool [17], and the Noncommunicating Children's Pain Checklist-Revised (NCCPC-R) [18]. Of these the r-FLACC is seen to have the most clinical utility for professionals [19], although even this tool cannot claim to be reliable and valid across all cognitively impaired children as it relies on typical behaviours, cues, and responses that some children may not express. Using a specific pain assessment tool that has been validated for use with children with cognitive impairment rather than a generic tool validated for nonimpaired children can increase the accuracy of pain assessment [20]. Despite these tools being readily available for use within practice, they are often not used routinely or with much enthusiasm by professionals partly due to lack of familiarity with the tools [1]. Professionals often rely on their own interpretation of a child's behaviour which can be variable as it can be swayed by their own attitudes and beliefs [15].

This paper reports part of a larger study that used a convergent parallel mixed method design [21] and which examined parent-reported pain experienced by children with profound cognitive impairment and parents' and healthcare professionals' experiences and perceptions of assessing and managing pain in this diverse group of children. This paper reports on data generated from the health professionals.

## 2. Methodology and Methods

We aimed to explore how healthcare professionals develop and acquire knowledge and skills to assess and manage pain in children with cognitive impairment.

### 2.1. Sampling

A mixture of purposive and snowball sampling aimed to recruit between 15 and 20 healthcare professionals with at least 6 months' experience of working with children with profound cognitive impairment from a single paediatric, tertiary referral centre in England. We aimed to ensure maximum variation of professionals in terms of their professional role, speciality, grade, and experience.

### 2.2. Interviews

Semistructured qualitative interviews allowed us to explore key areas of interest, for example, professionals' experiences of assessing and managing pain in children with complex needs, how they develop and acquire skills and knowledge managing pain in this patient group, and the meanings they attribute and/or associate with these experiences. Most of the interviews were conducted on hospital premises although some were undertaken by telephone. All interviews were undertaken at a time convenient to the healthcare professional during normal working hours. All interviews were audio-recorded and transcribed verbatim and transcripts anonymised.

### 2.3. Ethics

The study gained ethics approval via the NHS Research Ethics Service (14/NW/0106) and through the tertiary hospital. Informed consent was gained from each participant. A researcher who did not have any direct link to the hospital undertook the interviews. Care was also taken in relation to governance issues (e.g., anonymisation, data protection).

### 2.4. Data Analysis

We used thematic analysis in line with the approach advocated by Braun and Clarke [22]. Each member of the research team consisting of academic nurses and social scientists undertook analysis (coding and memoing) of selected interviews; the use of multiple coders aimed to promote the quality and rigour of analysis [23]. Discussion then took place within the team until a broad understanding and consensus about initial themes was achieved. From this point, two of the research team (Joan Simons & Bernie Carter) analysed all the 19 transcripts and used an iterative process of moving between transcripts and codes to identify emerging themes and attending to negative cases. Each participant's data were analysed as an individual dataset before considering all the transcripts as a complete dataset.

## 3. Findings

Within the findings, we firstly present a brief overview of the participants' demographics.

Nineteen health professionals participated in the study. Of these eight were working in nursing roles including assistant practitioner (*n* = 1), staff nurse (*n* = 3), and clinical nurse specialist (*n* = 4; three were specialists in pain, and one was a specialist in neurology). The five allied health professionals worked as an occupational therapist (*n* = 1), psychologist (*n* = 1), physiotherapist (*n* = 1), play specialist (*n* = 1), and movement therapist (*n* = 1). The medical professionals included anaesthetists (*n* = 2), neurologists (*n* = 2), general paediatrician (*n* = 1), and a pain specialist (*n* = 1). Of the 19 participants, 16 were female and three were male, and their experience of working with children ranged from two to more than 20 years. All worked within the tertiary hospital setting.

### 3.1. Themes

**Figure 1:**
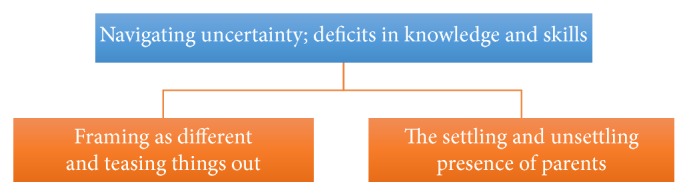
Metatheme and core themes.

The analysis resulted in the metatheme “navigating uncertainty; deficits in knowledge and skills” with two core themes “framing as different and teasing things out” and “the settling and unsettling presence of parents” (see Figure 1). In order to protect the identity of participants, anonymised quotations reported in the paper are identified as being from one of the three professional groups: nursing (N), medical (M), or allied health (AH).

### 3.2. Metatheme: Navigating Uncertainty; Deficits in Knowledge and Skills

The metatheme of “navigating uncertainty; deficits in knowledge and skills” encompasses the ways in which all the professionals, to a greater or lesser degree, felt challenged by aspects of assessing and managing the pain of children with cognitive impairment. Many of the professionals talked of feeling out of their comfort zone and feeling uncertain of their pain-related clinical decision-making. This uncertainty tended to erode professional confidence and many professionals discussed deficits in their skill and knowledge set. This uncertainty was expressed, despite demonstrating insight, knowledge, and understanding of children with profound cognitive impairment. None of the professionals talked of being able to undertake pain assessment in a completely fluid and intuitive way, explaining this was due to the idiosyncrasies they encountered with each child that prevented them from developing a reliable skill set. Although most were aware that pain assessment tools existed, they did not talk of these as a means of supporting their assessment or helping reduce uncertainty. Rather, uncertainty was managed through engaging with other people, most notably parents but also other professionals:

It becomes a dialogue really between the parents, the ward nurses and, well, the carers and ourselves (AH).

Dialogue across different disciplines and specialities, especially when the cause of pain and/or the most appropriate intervention were unclear, was seen to be important as this allowed different perspectives and solutions to be examined. The level of uncertainty perhaps reflects the fact that most learning was gained experientially,* “through experience and by discussing cases with colleagues and seniors” *(M). Learning was* “dripped in in different ways”* (AH) with very little formal training apart from some specific in-house sessions on a pain tool (the Paediatric Pain Profile). Most professionals stated they would welcome more education and training although many felt that this input should be clinical and not classroom oriented.

#### 3.2.1. Framing as Different and the Trickiness of Teasing Things out

As a group the professionals framed the children as being both very different to nonimpaired children and very different to each other. This was manifested in the professionals repeated references to the children being particularly unique, for example,* “every child [with cognitive impairment] really is different”* (M),* “every little thing they do is different”* (N), and they are* “really individual and unique” *(AH) when communicating their pain. The experiential knowledge and skills that professionals used when working with noncognitively impaired children did not seem to be accessible or transferrable when engaging with children with cognitive impairment. Professionals talked of this group of children being* “the trickiest patients”* (N) who were difficult to* “gauge”* (M) especially when the professional was* “not familiar”* with the individual child as often their engagement with children with cognitive impairment was* “very patchy”* (M).

Pain assessment was often described as being* “quite stressful …because they [child] can't tell you”* (N) although learning an individual child's responses and pain cues could be done* “over a period of time.”* Some professionals talked of having* “the luxury of having enough time… [and being able to] build a relationship”* (AH) with the parents and get to know the child; they were aware this was not possible for all professionals to achieve. The stress and the uncertainty associated with pain assessment were sometimes reported as making professionals think it was* “too difficult”* with the temptation to* “just refer the child on to someone else”* (N). However, as one of the experienced nurses explained* “you can't back away, can't ignore them”* (N).

Typical pain behaviours and responses usually relied on when working with nonimpaired children were seen as unreliable or inappropriate with cognitively impaired children. Not only were the children's pain cues individualistic but their responses to surgery or other interventions were reported to be more complex and unpredictable. Professionals talked of having to* “build up a picture… to really tease things out and help you focus in”* (AH) and looking out for* “those minor subtle differences from the way the child would normally behave”* (N).

“Teasing things out” was a* “skill [that] takes a while to develop”* (M) and involved the professionals drawing more strongly on observational skills, physical examination of the child, and considering the results of other investigations; again, they made little mention of pain assessment tools contributing to helping to tease things out. Some of the professionals were systematic in their approach:

My general approach is… history and examination first and foremost. So back to basics, trying to tease out through the history if it's a new symptom, what it is that's changed or what else is going on (M).

The professionals also acknowledged that they turned to the parents for input and often relied on the parents' knowledge of their child to help guide assessment, as one nurse explained:

…parents of children with complex needs are best placed to look at their needs, they are the ones that know how their child communicates so we would always listen to them in the first instance (N).

Professionals talked not only about the challenges inherent in assessment but also of how complex and tricky it was managing the children's pain. Depending on the cause of the pain, the pharmacological response tended to be multimodal and pharmacological intervention was described as* “a big minefield”* (N) that needed to be* “targeted and logical”* (M) even when there was uncertainty about the cause(s) of the pain and appropriate response(s). Professionals talked of the massive challenges associated with getting management right for the child and the need to* “have an in-depth knowledge about pain treatment and management that goes far beyond what I currently prescribe as painkillers” *(M). Therapies including physiotherapy and somatic therapy were identified as being helpful particularly when a* “broad angle approach using both passive and active therapy”* (AH) was implemented.

One way of trying to reduce the trickiness of pain management was the implementation of tailored pain plans for children as this helped to reduce uncertainty and create a greater sense of security for everyone involved in the child's care. Pain plans were perceived to be particularly important in terms of being a means of documenting a child's specific pain cues,* “treatment options and information about how to use different techniques”* (M), and* “a rescue plan for break out pain relief”* (N).

#### 3.2.2. The Settling and Unsettling Presence of Parents

Whilst professionals accepted it* “took time to develop”* (AH) good assessment skills, they often turned to parents for help in assessing their child's pain and determining a course of intervention, as one very experienced nurse explained:

I'll involve the parents and I'll say, ‘Do they normally do this? Is this what he normally does? Does he normally cry like that? Does he normally whinge [complain peevishly] like that? Does he ever get spasms? Is he on Baclofen already?' I find it really, really difficult to do it [assessment] by myself. (N)

Parental expertise and specialist knowledge of their child were often* “relied on …as they are the voice of the child” *(M), and some were acknowledged to have* “significant skills” *(AH), be “like a walking BNF [British National Formulary]” (N), and be* “good historians in that they'll tell you what they've had in the past and what has worked and what hasn't worked”* (M).

Whereas the professionals did not feel that they could rely on their own intuition as easily with this group of children, they recognised that very often the* “parents' intuition is second to none”* (N). However, there was also acknowledgement that what looked intuitive was often hard won and built up over time and parents were often quite systematic in their approach, as one doctor explained:

They go back and check systems in a semi systematic way. They know their child can be constipated so they give something for constipation. Or they realise if they move a leg in a certain way it hurts and if they put it in a different way, it gets better. Or they reposition them in the wheelchair …so they try things out (D).

Drawing on parental expertise to support clinical judgement was generally seen to be* “pretty vital”* and* “at the crux of it”* (AH), helping to reduce clinical uncertainty and the* “guesswork”* that professionals otherwise had to engage in. However, the professionals also cautioned against uncritically accepting parents as experts, noting that* “some parents may not want to think their child is in pain”* (AH) and* “some parents are less in tune”* (N). Genuine expertise was welcomed:

There's also a difference between the expert parent, who's expert in their child and there's the Google expert. When you have a genuine expert parent who's also pragmatic and sensible…it is wonderful (M).

When parents were expert and sensible and did not challenge professionals, their presence was seen to be settling. However, expert parents sometimes* “rocked the boat”* (N) by challenging decisions,* “shaking people's confidence,”* and appearing to* “be all knowing and powerful”* (N). This sort of behaviour shifted the balance from being a settling to unsettling presence on the ward. Whilst professionals acknowledged that the parents of children with cognitive impairment often had to* “fight for services”* (AH) and some were* “very direct and agree or disagree with assessment outcomes or opinions being given”* (AH), this was reported to be distressing for the professionals.

## 4. Discussion 

Uncertainty in clinical practice is a common phenomenon [24] and all professionals have to find ways of dealing with or navigating uncertain situations. To a greater or lesser extent, all of the professionals regardless of disciplinary background or years of experience expressed a sense of uncertainty about assessing and managing pain in children with profound cognitive impairment, describing it variously as tricky, complex, stressful, difficult, challenging, subtle, and unfamiliar. Their sense of uncertainty arose from feeling inadequately prepared, insufficiently knowledgeable, and unsure how to act in some situations. These descriptions and feelings align well with existing descriptions and definitions of uncertainty. Uncertainty is said to arise when “details of situations are ambiguous, complex, unpredictable, or probabilistic; when information is unavailable or inconsistent; and when people feel insecure in their own state of knowledge or the state of knowledge in general” (p478) [25].

Although uncertainty is part of everyday practice within clinical settings, many professionals profess a dislike for uncertainty [24, 26] and are most comfortable when working in more clinically certain situations, such as assessing pain in an articulate and verbal child. However, pain assessment in profoundly cognitively impaired children does not offer the security of a clinically certain situation; the professionals in our study found it challenging to navigate such situations. Their usual array of “navigation aids” such as experience of similar situations and asking more senior colleagues were reported as being less reliable, making them uncertain about assessment. They did not appear to consider turning to other potential navigation aids such as guidelines or assessment tools that could have provided scaffolding for their thinking [27, 28]. Although we did not explore why they did not turn to these scaffolds, there is considerable evidence that shows that personal factors (e.g., lack of familiarity with and awareness of guidelines, potential erosion of self-efficacy), external factors (e.g., organizational constraints, time restrictions), and guideline-related factors (e.g., complexity and unavailability) act as barriers to implementing guidelines into practice [27]. Brashers [25] proposes that uncertainty is difficult to deal with because it is multilayered and interconnected, meaning that the professionals' responses were often very contextually dependent; for example, the presence of parents could be both settling and unsettling. Parents were both a welcome settling presence (when information was requested, presented in an appropriate manner, and fitted the professional's frame of reference) and an unsettling presence (when the communication was perceived as undermining, directive, and underpinned by the wrong sort of expertise). Whilst uncertainty can produce positive feelings, our professionals tended to talk of uncertainty in terms of negative feelings such as anxiety or being overwhelmed. Similar findings have been shown in other studies examining clinical uncertainty [26]. Despite feeling out of their comfort zones none of our professionals talked of using avoidance, a tactic often used in situations of clinical uncertainty [26], as a means of removing themselves from the challenges inherent in pain assessment with children with profound cognitive impairment.

Uncertainty is self-perceived [25]. Thus, regardless of the wealth of knowledge, skills, and experience our professionals had to draw upon, their feelings of uncertainty framed the way they thought about and navigated their pain practice with children with profound cognitive impairment. One strategy for managing their uncertainty involved turning to people they perceived as being more experienced navigators with better expertise and/or more knowledge. Mostly this involved turning to parents for their child-centred wisdom and assessment skills, although they also turned to more senior or experienced professional colleagues for guidance. It was clear that our professionals were insightful about the limits to their knowledge and whilst it can be positive to “know what you don't know” [24], it can be limiting if the professionals focus on what they do not know. Focusing on knowledge deficits can be immobilising, resulting in professionals failing to draw on the knowledge they do have and which could be transferred. When the search for knowledge such as relevant contemporary guidelines or assessment tools becomes time-consuming then it acts as a barrier to action. Addressing perceived gaps in knowledge and skills through education, training, and mentorship may help reduce uncertainty by promoting greater confidence although as Hall [29] notes, it is impossible to completely remove uncertainty from decision-making. Effective knowledge translation strategies can help support health professionals and Stevens et al. [28] propose that the promotion of optimal pain practices is likely to need multiple, tailored knowledge transfer interventions and that these need to take into account organizational and contextual factors. Raising awareness of guidelines, tools, and local policy in relation to children with cognitive impairment is important. Creating an expectation that children with cognitive impairment will have their pain assessed using an appropriate and validated tool, auditing the implementation of the tool, and gaining and sharing feedback are strategies that could be employed in and across organisations. Such knowledge translation strategies could help to build the professionals' confidence in their knowledge and reduce their uncertainty in this important area of practice.

Whilst uncertainty may be unsettling for professionals, for the children who experience regular and ongoing pain, the impact of this uncertainty may be suboptimal pain management [2, 4, 7] resulting in the prolonged exposure to pain or the under- or overprescribing of pain medication. Inadequate pain assessment leaves a child vulnerable to their pain being missed or its severity unacknowledged [20]. This, in turn, impacts the effective treatment of a child's pain with the most likely outcome being undertreatment.

The findings of this study are limited in several ways. The study was undertaken in one children's health care setting and thus may have limited generalizability. Our use of snowball sampling may have limited the diversity of participants although this was not apparent as our participants came from a range of different settings and disciplinary backgrounds. We were not prescriptive in asking professionals to focus on a specific, perhaps more homogenous group of children with profound cognitive impairment, so our findings could be critiqued as being generic. The use of interviews provided the professionals with the opportunity to openly explore their experience of practice but it may have inhibited them from revealing incidents of poor practice or avoidant behaviour that might be expected where high levels of uncertainty are reported.

## 5. Conclusion

Navigating uncertainty in the assessment and management of pain in children with profound cognitive impairment was clearly an issue for professionals in the study. Yet despite this, it was evident that they were “teasing things out” and trying to unravel a complex and tricky situation to ensure that the children received good pain care.

Navigating the complexity of identifying and managing pain appropriately in children with profound cognitive impairment is amplified by variations in how each child experiences and expresses their pain. Most professionals recognised parents as being expert navigators of their own child's idiosyncratic behaviours and responses to pain and often deferred to parents for advice. However, this was not consistent and parents' involvement in the management of pain in their children was sometimes perceived as challenging by professionals.

Reducing the challenges of navigating uncertainty may involve better formal education and training opportunities for professionals so as to address some of the ambiguity, complexity, and insecurity that professionals currently face. However, given the challenge of “teasing things out” and managing pain in children with profound cognitive impairment and the complexity of developing expertise in the pain profile of individual children, consideration should be given to developing pain assessment and management practice that is inclusive and involves both parents and practitioners and supports and enables parents to be active participants in the process.
